# Apocynin improving cardiac remodeling in chronic renal failure disease is associated with up-regulation of epoxyeicosatrienoic acids

**DOI:** 10.18632/oncotarget.5084

**Published:** 2015-08-17

**Authors:** Kun Zhang, Yu Liu, Xiaoqiang Liu, Jie Chen, Qingqing Cai, Jingfeng Wang, Hui Huang

**Affiliations:** ^1^ Department of Cardiology, Sun Yat-Sen Memorial Hospital, Sun Yat-Sen University, Guangzhou 510120, China; ^2^ Guangdong Province Key Laboratory of Arrhythmia and Electrophysiology, Guangzhou 510120, China; ^3^ Department of Cardiology, the People's Hospital of Guangxi Zhuang Autonomous Region, Nanning 530000, China; ^4^ Department of Clinical laboratory, Sun Yat-Sen Memorial Hospital, Sun Yat-Sen University, Guangzhou 510120, China; ^5^ Department of Radiation Oncology, Sun Yat-Sen Memorial Hospital, Sun Yat-Sen University, Guangzhou 510120, China; ^6^ Department of Medical Oncology, Sun Yat-Sen University Cancer Center, Sun Yat-Sen University, Guangzhou 510120, China

**Keywords:** Pathology Section, epoxyeicosatrienoic acids, chronic renal failure, cardiac remodeling, apocynin

## Abstract

Cardiac remodeling is one of the most common cardiac abnormalities and associated with a high mortality in chronic renal failure (CRF) patients. Apocynin, a nicotinamide-adenine dinucleotide phosphate (NADPH) oxidase inhibitor, has been showed cardio-protective effects. However, whether apocynin can improve cardiac remodeling in CRF and what is the underlying mechanism are unclear. In the present study, we enrolled 94 participants. In addition, we used 5/6 nephrectomized rats to mimic cardiac remodeling in CRF. Serum levels of epoxyeicosatrienoic acids (EETs) and its mainly metabolic enzyme-soluble epoxide hydrolase (sEH) were measured. The results showed that the serum levels of EETs were significantly decreased in renocardiac syndrome participants (*P* < 0.05). In 5/6 nephrectomized CRF model, the ratio of left ventricular weight /body weight, left ventricular posterior wall thickness, and cardiac interstitial fibrosis were significantly increased while ejection fraction significantly decreased (*P* < 0.05). All these effects could partly be reversed by apocynin. Meanwhile, we found during the process of cardiac remodeling in CRF, apocynin significantly increased the reduced serum levels of EETs and decreased the mRNA and protein expressions of sEH in the heart (*P* < 0.05). Our findings indicated that the protective effect of apocynin on cardiac remodeling in CRF was associated with the up-regulation of EETs. EETs may be a new mediator for the injury of kidney-heart interactions.

## INTRODUCTION

Cardiac remodeling has been demonstrated to be a common cardiac abnormality in chronic renal failure (CRF) patients and associates with a high cardiovascular mortality [1]. It is showed that about 70%–80% end-stage renal disease (ESRD) patients have manifestation of left ventricular hypertrophy (LVH) [2]. Although hemodialysis and peritoneal dialysis are effective replacement therapies for improving the prognosis of CRF patients, cardiovascular diseases are still the leading cause of death. So to find out the key mediator and therapeutic strategies remains urgent.

Epoxyeicosatrienoic acids (EETs) are the products of arachidonic acids (AA) metabolized by cytochrome P450 (CYP) epoxygenases. Our and previous studies demonstrated that EETs could reverse cardiac remodeling [3, 4]. However, it is still lack of direct evidence to show that EETs could improve cardiac remodeling in CRF, although we reviewed the possible mechanisms of EETs to improve cardiac remodeling in CRF disease [5]. In addition, previous studies mainly focus on the therapeutic effects of EETs, whether EETs are a key mediator for both cardiac and renal injury is still unknown.

Apocynin (4-hydroxy-3-methoxyacetophenone), an inhibitor of nicotinamide-adenine dinucleotide phosphate (NADPH) oxidase, has been demonstrated to attenuate cardiac remodeling through reducing oxidative stress [6, 7]. In fact, apocynin is not a special inhibitor of NADPH, it has role of anti-inflammatroy, reducing blood pressure, ameliorating neuronal death, improving renal injury, etc [8–11]. However, whether it has the effect on regulating the metabolism of EETs and improves cardiac remodeling in CRF are unclear. In the present study, we hypothesized that apocynin could improve cardiac remodeling in CRF and this effect may be associated with the metabolism of EETs. EETs may be an important mediator for the injury of kidney-heart interactions.

## RESULTS

### Demographic and clinical characteristics of the enrolled participants

A total of 94 participants were enrolled in this study. According to the both renal and heart function, we grouped the participants in to normal group, renal dysfunction (RD) group, heart dysfunction (HD) group, and renocardiac syndrome (RCS) group. The demographic and clinical characteristics were showed in Table 1. As seen from the table, the RCS group had higher levels of serum creatinine, blood urea nitrogen (BUN), N-terminal pro-brain natriuretic peptide (NT-proBNP), uric acid, and C-reactive protein (CRP) than normal, RD and HD groups. Simultaneously, the RCS group had lower estimated glomerular filtration rate (eGFR), ejection fraction (EF), superoxide dismutase (SOD), albumin, and hemoglobin than other three groups. Moreover, the significant overall differences were also found in age, the serum levels of sodium, chorine, low density lipoprotein cholesterol (LDL-c), left atrium (LA), and left ventricular end-diastolic dimension (LVEDD) (*P* < 0.05, Table 1).

**Table 1:** The characteristics of enrolled participants

Characteristics	Normal (*n* = 23)	RD (*n* = 25)	HD (*n* = 24)	RCS (*n* = 22)	*P* value
**Baseline Characteristics**					
Age (year)	51.17 ± 1.34	64.27 ± 2.04^a^	68.59 ± 1.60^a^	72.00. ± 2.52^a^	<0.001
Sex (Male/Female)	13/10	14/11	12/12	12/10	–
SBP (mmHg)	125.35 ± 3.52	138.33 ± 3.27	128.61 ± 5.64	132.50 ± 5.19	0.073
DBP (mmHg)	77.65 ± 2.16	80.13 ± 1.93	77.28 ± 2.88	79.75 ± 2.92	0.58
Hear rate (b.p.m)	76.52 ± 3.01	77.60 ± 3.04	86.13 ± 2.88	86.10 ± 2.72	0.13
**Biochemical Characteristics**					
K (mmol/L)	3.90 ± 0.072	4.08 ± 0.076	3.82 ± 0.064	3.91 ± 0.13	0.24
Na (mmol/L)	141.41 ± 0.57	140.29 ± 0.46	137.23 ± 1.54^a^^b^	137.66 ± 1.07^a^^b^	0.006
Cl (mmol/L)	104.42 ± 0.61	104.22 ± 0.56	100.91 ± 1.74^a^^b^	99.69 ± 1.02^a^^b^	0.001
Ca (mmol/L)	2.27 ± 0.025	2.23 ± 0.022	2.15 ± 0.14	2.17 ± 0.027	0.12
Mg (mmol/L)	0.88 ± 0.023	1.29 ± 0.45	0.80 ± 0.039	0.80 ± 0.032	0.087
Fe (μmol/L)	21.32 ± 7.39	15.85 ± 1.54	10.25 ± 2.16	13.15 ± 1.70	0.28
GLU (mmol/L)	4.86 ± 0.12	5.68 ± 0.31	5.14 ± 0.89	5.21 ± 0.21	0.28
TC (mmol/L)	4.92 ± 0.15	4.75 ± 0.17	4.25 ± 0.31^a^	4.12 ± 0.24^a^	0.058
Triglycerides (mmol/L)	1.52 ± 0.20	1.80 ± 0.22	1.29 ± 0.11	1.58 ± 0.14	0.37
HDL-c (mmol/L)	1.42 ± 0.072	1.15 ± 0.058	1.02 ± 0.16	1.21 ± 0.11	0.098
LDL-c (mmol/L)	2.96 ± 0.15	2.66 ± 0.12	2.42 ± 0.14^a^	2.22 ± 0.27^a^	0.015
Albumin (g/L)	41.78 ± 0.68	40.74 ± 1.76	38.51 ± 0.78	35.45 ± 0.98^a^	0.22
Creatinine (μmol/L)	61.04 ± 2.20	200.29 ± 7.40^a^	102.72 ± 3.4^b^	278.23 ± 37.74^a^^c^	<0.001
BUN (mmol/L)	5.00 ± 2.58	9.92 ± 1.13^a^	7.23 ± 0.57^b^	13.83 ± 1.31^a^^b^^c^	<0.001
UA (μmol/L)	310.84 ± 15.00	430.41 ± 20.79^a^	427.22 ± 23.57^a^	543.92 ± 39.46^a^^b^^c^	<0.001
NT-proBNP (pg/ml)	43.4 ± 9.83	7370.56 ± 1987.24^a^	11624.56 ± 1388.49^a^	16303.57 ± 4280.70^a^^b^	<0.001
CRP (mg/L)	2.54 ± 0.71	33.77 ± 12.96^a^	57.10 ± 12.27^a^	70.61 ± 24.80^a^^b^	0.044
SOD (U/ml)	124.36 ± 2.47	108.80 ± 2.86^a^	103.12 ± 3.72^a^	88.50 ± 7.84^a^^b^	<0.001
Hb (g/L)	135.04 ± 2.19	121.85 ± 2.95^a^	130.56 ± 8.12^b^	113.92 ± 7.4^a^^c^	0.026
eGFR (ml/min.1.73m^2^)	105.10 ± 2.09	47.91 ± 3.94^a^	63.77 ± 2.48^a^^b^	30.61 ± 4.76^a^^b^^c^	<0.001
**Echocardiographic Characteristics**					
LA (mm)	29.87 ± 0.73	34.39 ± 0.79^a^	39.08 ± 0.76^a^^b^	37.62 ± 1.29^a^	<0.001
IVSd (mm)	9.65 ± 1.40	10.61 ± 0.40	9.39 ± 0.65	10.15 ± 0.60	0.67
LVEDD (mm)	45.30 ± 0.67	49.29 ± 0.92	58.73 ± 1.42^a^^b^	55.54 ± 3.77^a^^b^	<0.001
LVPWd (mm)	8.22 ± 0.24	10.62 ± 0.38	10.25 ± 0.77	10.15 ± 0.44	0.20
EF%	67.26 ± 0.89	63.27 ± 1.09	36.69 ± 1.24^a^^b^	40.00 ± 2.32^a^^b^	<0.001

All values are expressed as mean ± SEM.

*P*-value represents the comparison among groups using least-significant difference (LSD) test.

a *P* < 0.05, (RD, HD, RCS) group vs. Normal group

b *P* < 0.05, (HD, RCS) group vs. RF group

c *P* < 0.05, RCS group vs. HD group

Abbreviations: BUN, blood urea nitrogen; Ca, calcium; Cl, chloride; CRP, C-reactive protein; DBP, diastolic blood pressure; EETs, epoxyeicosatrienoic acids; EF, ejection fraction; eGFR, estimated glomerular filtration rate; Fe, iron; GLU, glucose; Hb, hemoglobin; HD, heart dysfunction; HDL-c, high density lipoprotein cholesterol; IVSd, interventricular septum depth; K, potassium; LA, left atrium; LDL-c, low density lipoprotein cholesterol; LVEDD, left ventricular end-diastolic dimension; LVPWd, left ventricular posterior wall depth; M/F, male/female; Mg, magnesium; Na, sodium; NT-proBNP, N-terminal pro-brain natriuretic peptide; RCS, renocardiac syndrome; RD, renal dysfunction; SBP, systolic blood pressure; SOD, superoxide dismutase; TC, total cholesterol; UA, uric acid.

### The levels of 14, 15-dihydroxyeicosatrienoic acid (DHET) decreased with reduced renal function

**Figure 1:**
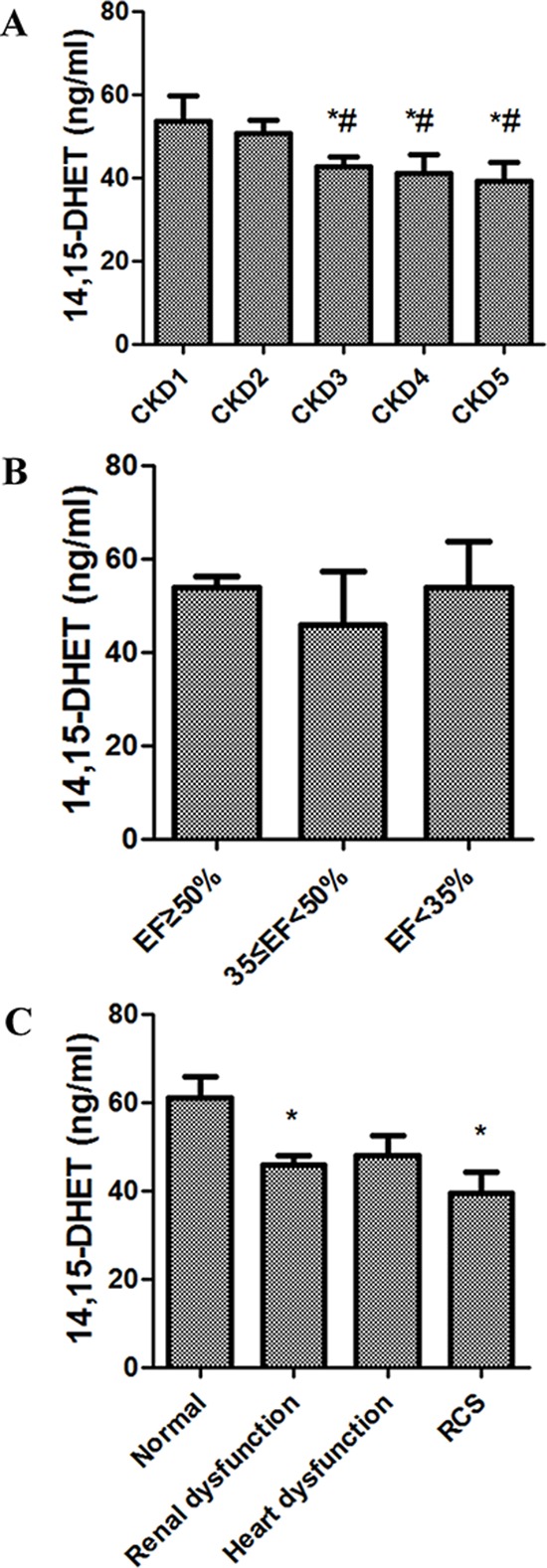
The levels of 14,15-DHET in patients with chronic kidney disease **A.** The levels of 14,15-DHET in different CKD groups. We grouped the participants into five CKD stages: CKD 1 (eGFR ≥ 90 ml/min), CKD 2 (89 ≥ eGFR ≥ 60 ml/min), CKD 3 (59 ≥ eGFR ≥ 30 ml/min), CKD 4 (29 ≥ eGFR ≥ 15 ml/min), and CKD 5(eGFR < 15 ml/min). The levels of 14,15-DHET significantly decreased in CKD stage 3 to 5 groups (*P* < 0.05). DHET, dihydroxyeicosatrienoic acid; CKD, chronic kidney disease. *P < 0.05 *vs*.CKD1 group; *P < 0.05 *vs*.CKD2 group **B.** The levels of 14,15-DHET in different cardiac function groups. we divided the participants with no or little kidney damage (CKD stage 1 and 2) into three different cardiac function groups depending on EF: group 1, EF ≥ 50%; group 2, 50> EF ≥ 35%; group 3, EF < 35%. No significant difference of the levels of 14,15-DHET was found in different cardiac function groups (*P* > 0.05). DHET, dihydroxyeicosatrienoic acid; CKD, chronic kidney disease. **C.** The levels of 14,15-DHET in different renal and cardiac function groups. According to both the renal and cardiac functions, four groups were divided: normal, renal dysfunction, heart dysfunction, and RCS groups. The levels of 14,15-DHET significantly reduced in renal dysfunction and RCS groups (*P* < 0.05). DHET, dihydroxyeicosatrienoic acid; RCS, renocardiac syndrome. *P < 0.05 *vs.* Normal group

Depending on the eGFR, we grouped the participants into five chronic kidney disease (CKD) groups: CKD 1 (eGFR ≥ 90 ml/min, *n* = 35), CKD 2 (89 ≥ eGFR ≥ 60 ml/min, *n* = 13), CKD 3 (59 ≥ eGFR ≥ 30 ml/min, *n* = 24), CKD 4 (29 ≥ eGFR ≥ 15 ml/min, *n* = 9), and CKD 5 (eGFR < 15 ml/min, *n* = 13). We found that CKD3 to CKD5 groups had significant lower levels of 14, 15-DHET comparing with that in CKD1 and CKD2 groups (*P* < 0.05). However, no significant difference was found among these three groups (Figure 1A). Similarly, to exclude the influence of renal function, and explore the role of cardiac function, we divided the participants with no or little kidney damage (CKD stage 1 and 2) into three different cardiac function groups depending on EF: group 1 (EF ≥ 50%, *n* = 38), group 2 (50% > EF ≥ 35%, *n* = 19), and group 3 (EF < 35%. *n* = 17). It was showed that the levels of 14, 15-DHET decreased at the early stage of HD and a slightly increase at the final stage of HD. However, no significance was found among groups (*P* > 0.05) (Figure 1B). Then we investigated the combined effects of renal and cardiac functions on the levels of 14, 15-DHET, the results showed that comparing with the normal, RD and HD groups, the levels of 14, 15-DHET were further reduced in RCS group (Figure 1C).

### Apocynin improved cardiac remodeling in nephrectomized (NE) CRF model

To mimic the cardiac remodeling in CRF, NE CRF model was used. As presented in Table 2, after 8 weeks surgery, the serum creatinine, the ratio of left ventricular weight / body weight (LVM/BW), and the left ventricular posterior wall thickness at diastole (LVPWd) were significantly increased while the EF was significantly reduced in NE group compared with that of sham-operated group (*P* < 0.05). It indicated the renal function was significantly decreased and there exited an obvious cardiac remodeling in NE group. Moreover, comparing with sham-operated group, the systolic blood pressure (SBP), and diastolic blood pressure (DBP) were also greatly elevated in NE group (*P* < 0.05). Interestingly, we found all the above effects could be improved by apocynin treatment except serum creatinine (Table 2). However, no significant difference of heart rate was found among groups.

**Table 2:** Characteristics in different treatment groups at 8 weeks post-surgery

	Sham-operated (*n* = 10)	NE (*n* = 10)	NE+apocynin (*n* = 10)
Body weight (g)	360 ± 24	259 ± 20^a^	290 ± 23^a,b^
SBP (mmHg)	118.71 ± 4.79	162.20 ± 2.77^a^	145 ± 3.22^a,b^
DBP (mmHg)	95.00 ± 6.38	110.60 ± 16.89^a^	98.21 ± 5.43^b^
HR (b.p.m)	317.57 ± 44.14	334.80 ± 31.14	325 ± 23.87
Creatinine (mmol/L)	54.50 ± 5.80	139.80 ± 32.31^a^	135.45 ± 7.82^a^
LV weight (g)	0.59 ± 0.05	0.82 ± 0.05^a^	0.65 ± 0.03^a,b^
LV weight/body weight (g/kg)	1.70 ± 0.13	3.20 ± 0.21^a^	2.14 ± 0.16^a,b^
LVPWd (mm)	1.13 ± 0.03	1.36 ± 0.09^a^	1.25 ± 0.07^a,b^
LVEDD (mm)	7.48 ± 0.49	7.685 ± 0.62	7.49 ± 0.43
LVESD (mm)	4.87 ± 0.33	5.98 ± 0.35^a^	5.07 ± 0.24^b^
EF%	65.1 ± 2.4	49.2 ± 4.7^a^	61.1 ± 4.4^b^

Abbreviations: NE, 5/6 nephrectomized; LV, left ventricular; LVPWd, left ventricular posterior wall thickness at diastole; LVEDD, left ventricular end-diastolic diameter; LVESD, left ventricular end-systolic diameter; EF, ejection fraction.

Data were expressed as mean ± SD.

a *P* < 0.05 vs. sham-operated group;

b *P* < 0.05 vs. NE group

**Figure 2:**
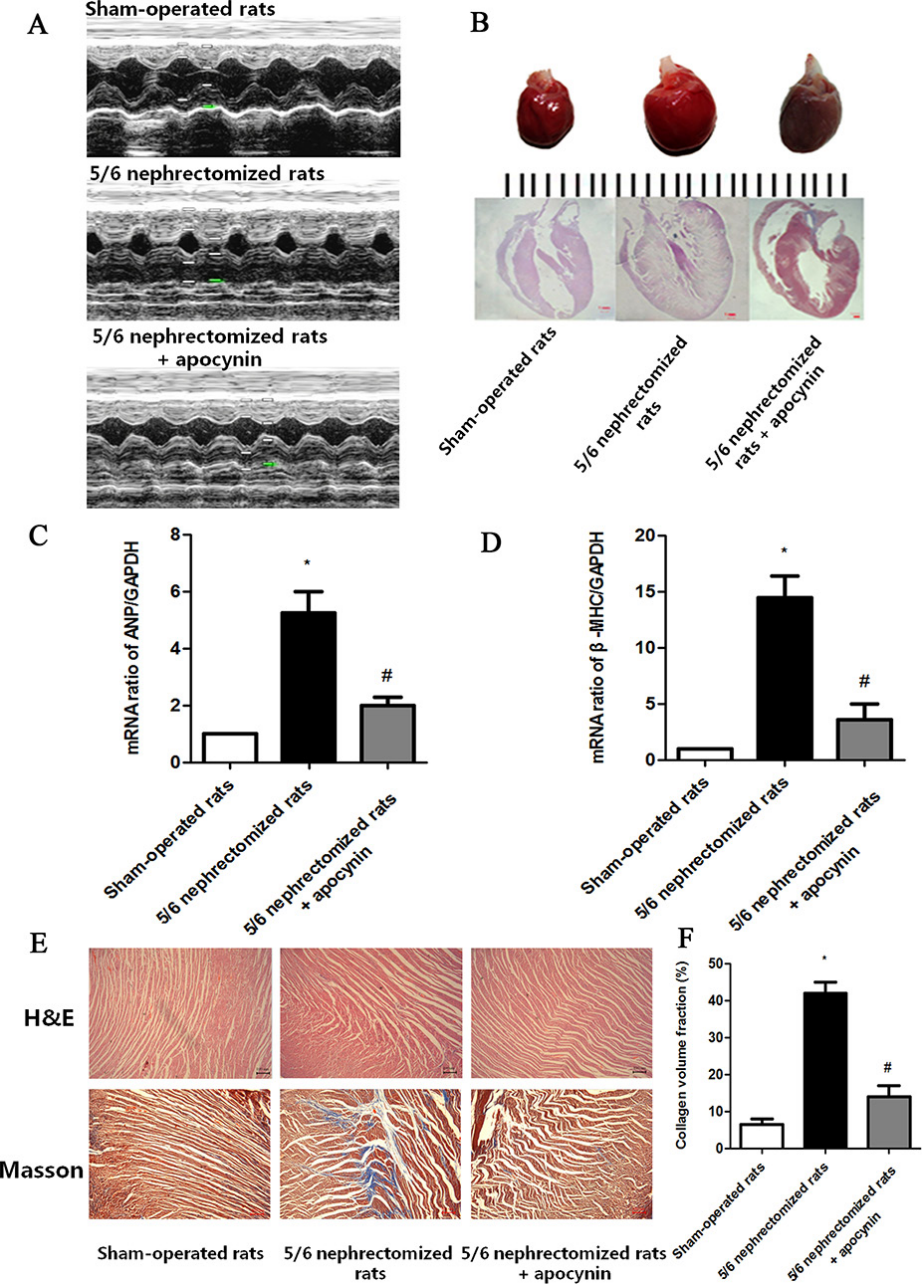
Apocynin improved cardiac hypertrophy and cardiac fibrosis in 5/6 nephrectomized group **A.** Echocardiogram data showed that apocynin decreased the 5/6 nephrectomized induced increase of left ventricular wall thickness. **B.** Apocynin reduced the 5/6 nephrectomized induced increase of heart size. **C.** Apocynin decreased the 5/6 nephrectomized induced elevation of mRNA expressions of ANP. ANP, atrial natriuretic peptide; *P < 0.05 *vs.* sham-operated group; *P < 0.05 *vs.* nephrectomized group. **D.** Apocynin decreased the 5/6 nephrectomized induced elevation of mRNA expressions of β-MHC. β-MHC, β-myosin heavy chain. *P < 0.05 *vs.* sham-operated group; *P < 0.05 *vs.* nephrectomized group. **E.** Hematoxylin and eosin staining and Masson trichrome staining showed that apocynin decreased the 5/6 nephrectomized induced increase of cardiac interstitial fibrosis. **F.** Apocynin decreased the 5/6 nephrectomized induced increase of cardiac collagen volume fractions. The cardiac collagen volume fractions were calculated as the ratio of aniline blue-stained fibrosis areas to total myocardium areas. *P < 0.05 *vs.* sham-operated group; *P < 0.05 *vs.* nephrectomized group.

Figure 2A-2F further showed the cardiac remodeling (cardiac hypertrophy and cardiac fibrosis, respectively) in different treatment groups. It was found that there had an obvious concentric hypertrophy (Figure 2A-2B) and cardiac interstitial fibrosis (Figure 2E) in NE group. And the cardiac hypertrophy markers atrial natriuretic peptide (ANP) and beta myosin heavy chain (β-MHC) and cardiac fibrosis index cardiac collagen volume fractions were also significantly increased compared with sham-operated group (Figure 2C, 2D, 2F). However, all the effects of promoting cardiac hypertrophy and cardiac fibrosis could be reversed by apocynin (Figure 2A-F).

### Apocynin decreased the expressions of cardiac soluble epoxide hydrolase (sEH) and increased the circulating levels of 14, 15-DHET in NE CRF model

**Figure 3:**
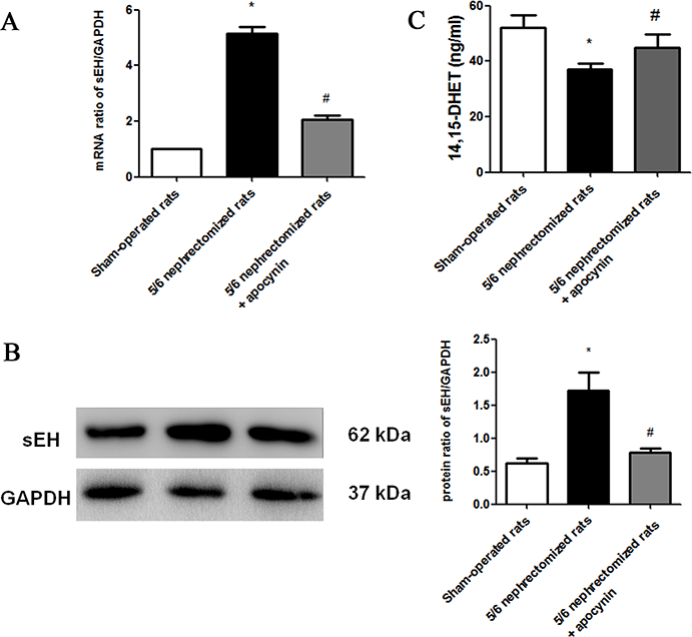
Apocynin reduced the 5/6 nephrectomized induced elevation of cardiac sEH in mRNA A. and protein B. levels **C.** showed that apocynin increased the 5/6 nephrectomized induced reduction of serum levels of *P < 0.05 *vs.* sham-operated group; *P < 0.05 *vs.* nephrectomized group. 14,15-DHET. DHET, dihydroxyeicosatrienoic acid; sEH, soluble epoxide hydrolase.

Figure 3A and Figure 3B showed the expressions of cardiac sEH in mRNA and protein levels. Both results indicated that the expressions of cardiac sEH significantly increased and apocynin prevented the up-regulation of sEH in CRF. Moreover, we measured the serum levels of 14, 15-DHET in different groups. It was showed that the serum levels of 14, 15-DHET significantly decreased in CRF while apocynin partly inhibited this effect (Figure 3C).

### Apocynin inhibited angiotensin II (Ang II) induced expressions of sEH in H9c2 cardomyocytes

**Figure 4:**
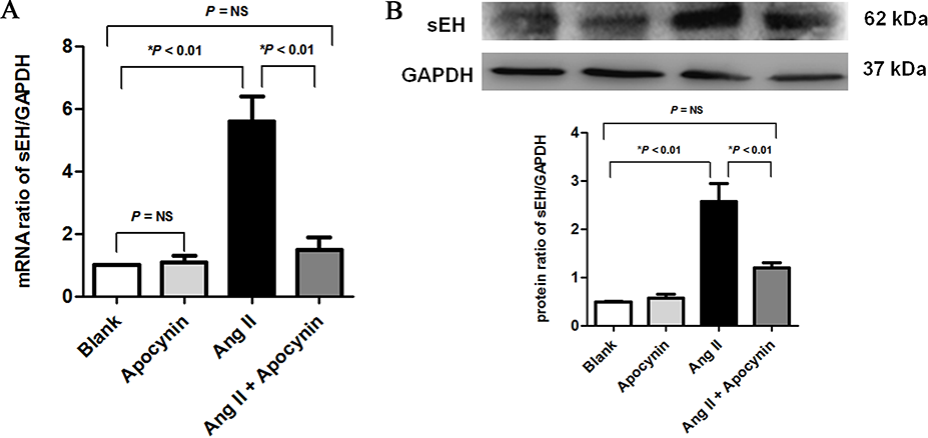
Apocynin inhibited the angiotensin II (Ang II) induced increased expressions of sEH both in mRNA A. and protein B. levels in H9c2 cardomyocytes sEH, soluble epoxide hydrolase. NS, no significance.

To further demonstrate the influence of apocynin on the expressions of sEH, we treated H9c2 cardomyocytes with Ang II. The results also showed that apocynin could decrease the mRNA and protein expressions sEH *in vitro* (Figure 4A-4B).

## DISCUSSION

The current study explored the role of EETs during the process of apocynin improving cardiac remodeling in CRF. We found that the levels of EETs decreased with the reduced renal function (CKD stage 3 to 5). And apocynin could improve cardiac remodeling accompanied with up-regulating the levels of EETs and inhibiting the cardiac expressions of sEH in CRF.

In CRF, the cardiac injury commonly exists and this phenomenon is called RCS (Type 4 cardiorenal syndrome) [12]. Oxidative stress, inflammation, renin-angiotensin-aldosterone system (RAAS) over activation, insulin resistance, etc are all considered to be the important mechanisms for the kidney-heart interactions [5, 13, 14]. And in clinic, the drugs administration such as angiotensin-converting enzyme inhibitors (ACEI), angiotensin receptor blockers (ARB) are also targeted to these mechanisms. Apocynin, a commonly use inhibitor of NADPH oxidase, can reduce oxidative stress induced by angiotensin II, tumor necrosis factor-α (TNF-α), etc [13, 15]. So it is widely used in studies for its antioxidation. Actually, apocynin is not a special NADPH oxidase inhibitor, it also has antihypertensive effect and anti-inflammatory [16, 17]. However, whether apocynin can regulate the metabolism of EETs in CRF has not been studied before. In the present study, we found apocynin could improve cardiac remodeling in CRF and this effect was associated with up-regulating the circulating levels of EETs and decreasing the cardiac expressions of sEH. It indicates that besides the antioxidation, regulating the metabolism of EETs maybe a new underlying mechanism for apocynin improving cardiac remodeling in CRF. And it also importantly implies that EETs maybe new mediators for the kidney-heart interactions.

EETs are the CYP-derived products of AA. There are four EET regioisomers: 5, 6-EET, 8, 9-EET, 11, 12-EET and 14, 15-EET [18]. Once EETs are formed, they are quickly converted to the corresponding DHETs by sEH. Elmarakby AA *et al*. and we both reviewed the therapeutic role of EETs in kidney and heart [5, 19]. And EETs also showed the mediator role for kidney-heart interactions. There are several possible explanations for this viewpoint. First, EETs were mainly produced by endothelial cells [20]. EETs were identified as endothelium-derived hyperpolarizing factors (EDHF) inducing dilation of renal and coronary arteries [21, 22]. And this effect was associated with lowering blood pressure which is a major determinant of cardiac remodeling in CRF [23]. Second, inflammation, RAAS over activation, oxidative stress, etc are all exist under RCS condition [23] and all these factors participates in influencing the production of EETs [24–26]. Third, EETs have autocrine and paracrine effects both in cardiovascular and renal systems [27]. In CRF, the uremic toxins may influence the affinity for EETs to bind to its receptor or inhibit its intracellular actions. However, whether other possibilities can good explain the mediator role of EETs for kidney-heart interactions are still need to explore.

There are several limitations of the present study. First, *in vitro*, the angiotensin II induced cardiac remodeling model cannot completely mimic the CRF condition, since a series of mechanisms are disorder in CRF. Second, apocynin is an inhibitor of NADPH. So cardiac remodeling reversed by apocynin in CRF was partly attributed to its antioxidation [28]. Third, we only used serum creatinine to evaluate renal function. Combing other good markers such as albuminuria may be better to evaluate kidney injury in CKD. Furthermore, in this study, we did not explore the mechanism that EETs influence the kidney-heart interactions.

In conclusion, the levels of EETs were decreased in CKD stage 3 to 5, especially in RCS patients. The protective effect of apocynin on cardiac remodeling in CRF was associated with the up-regulation of EETs. EETs may be a new mediator for kidney-heart interactions.

## MATERIALS AND METHODS

### Study population

This study protocol conformed to the ethical guidelines of the 1975 Declaration of Helsinki as reflected in *a priori* approval by the Ethics Committee of Sun Yat-sen University.

Between January 2013 and Jun 2014, a total of 94 participants at Sun Yat-sen Memorial Hospital of Sun Yat-sen University were initially enrolled in this study. 23 were the healthy volunteers, and 71 participants were patients. The healthy controls were recruited from the health screening center of our hospital. They were selected according to their self-reported medical history based on their age and gender distribution. The patients all had chronic renal and/or heart dysfunction. Besides the symptom and signs, RD was mainly evaluated by eGFR while the HD was mainly evaluated by echocardiography. The exclusion criteria were as follows: age < 18 years, acute cardiovascular and cerebrovascular ischemia diseases, acute renal failure, kidney transplantation, severe infection, severe hepatopathy, malignant tumor. According to both the renal and cardiac functions, four groups were divided: normal, RD, HD, and RCS groups. Moreover, depending on the eGFR, we also grouped the participants into five CKD stages: CKD 1 (eGFR ≥ 90 ml/min), CKD 2 (89 ≥ eGFR ≥ 60 ml/min), CKD 3 (59 ≥ eGFR ≥ 30 ml/min), CKD 4 (29 ≥ eGFR ≥ 15 ml/min), and CKD 5(eGFR < 15 ml/min). To exclude the influence of renal function, and explore the role of cardiac function, we divided the participants with no or little kidney damage (CKD stage 1 and 2) into three different cardiac function groups depending on EF: group 1, EF ≥ 50%; group 2, 50> EF ≥ 35%; group 3, EF < 35%.

### Biochemical data collection and EETs measurements

Blood samples from humans or Rats were drawn and transferred into tubes containing ethylenediaminetetraacetic acid (EDTA) and then stored at −20°C before being analyzed. Serum potassium, sodium, calcium, chloride, magnesium (Mg), BUN, iron (Fe), glucose, hemoglobin, albumin, uric acid, triglyceride, total cholesterol (TC), LDL-c, high density lipoprotein cholesterol (HDL-c), CRP, NT-proBNP, and SOD were measured using TBA-120 auto-analyzer (Toshiba Medical Systems, Japan).

Plasma levels of EETs were determined by gas chromatographymass spectrometry with negative-ion chemical-ionization using 14, 15-EETs/DHET Elisa kit (Detroit R&D) [29]. The changed levels of 14, 15-DHET indirectly reflects the changes of the levels of 14, 15-EETs.

### Animal model of CKD

Animal experiments were approved by the Committee on Ethics of Animal Experiments, Sun Yat-sen University and conducted in accordance with the Guidelines for the Care and Use of Laboratory Animals published by the US National Institutes of Health (NIH Publication No. 85–23, revised 1996).

Male Sprague-Dawley (SD) rats weighing 160–180g were obtained from Sun Yat-sen University. A two-step 5/6 NE model of CRF was used as described in our previous study [28]. Rats were randomly divided into three groups: sham-operated group, NE group and NE+apocynin group. Rats in NE+apocynin group were fed with 1.5 mM apocynin (Sigma-Aldrich, St. Louis, US) in drinking water [30]. 8 weeks after surgery, SBP, DBP and heart rate of the animals were measured by a tail-cuff method (BP-98A, Softron, Tokyo, Japan). Plasma was obtained by tail vein at 8 weeks for determining the levels of creatinine (model 7600–010, HITACHI automatic analyzer, Tokyo, Japan) and 14, 15-EET levels (Detroit R&D Inc.). Cardiac tissues were harvested at sacrifice for histological and molecular investigations.

### Echocardiography

Echocardiography was performed at 8 weeks post surgery using M-mode and two-dimensional measurements as described previously [31]. LA, LVPWd, LVEDD, left ventricular end-systolic diameter (LVESD), interventricular septum depth (IVSd) and EF were measured.

### Histological analysis

After harvest, the hearts were fixed overnight in 4% paraformaldehyde and then embedded in paraffin. Tissue sections of 4 μm thickness were prepared and stained with hematoxylin/eosin (HE) and Masson trichrome (Beyotime institute of Biology, Suzhou, China). The cardiac collagen volume fractions were calculated as the ratio of aniline blue-stained fibrosis areas to total myocardium areas with Image Pro-plus 5.0 software (Mediator Cybernetics, Bethesda, US).

### Cell culture and treatments

The H9c2 rat cell line was obtained from American Type Culture Collection (ATCC) (Sanger Biotech, Shang Hai, China). Cells were cultured in standard Dulbecco's modified Eagle's medium (DMEM) supplemented with 10% fetal bovine serum in a humidified incubator with 5% CO2 at 37°C. Cells were cultured in serum-free DMEM for 24 h before treatment. Four groups were divided: (a) dimethyl sulfoxide (DMSO) alone, (1 μl, Sigma-Aldrich, St. Louis, US) (b) apocynin (100 μM) alone (c) Ang II (100 nM, Sigma-Aldrich, St. Louis, US) (d) Ang II (100 nM)+apocynin (100 μM).

### Quantification by real-time polymerase chain reaction (PCR)

Total RNA was isolated from hearts or H9c2 cells. Quantitative real-time polymerase chain reaction (RT-PCR) was performed as previously described [3]. The nucleotide sequences of the primers were shown in Table 3.

**Table 3:** Prime sequences used in real-time polymerase chain reaction (PCR)

Gene	Primer sequence	Length
ANP	Forward: ATCTGATGGATTTCAAGAACC Reverse: CTCTGAGACGGGTTGACTTC	169bp
beta-MHC	Forward: CCTCGCAATATCAAGGGAAA Reverse: TACAGGTGCATCAGCTCCAG	198bp
sEH	Forward: AAGCCTGTGGAGCCAGTCTA Reverse: CCAGTTGTTGGTGACAATGC	185bp
GAPDH	Forward: ACTCCACGACATACTCAGCAC Reverse: CATCAACGACCCCTTCATT	197bp

### Western blot analysis

Heart tissues or H9c2 cells were homogenized in 250 μ of homogenization buffer using an electronic stirrer. Protein concentration was determined with BCA kit (Biocolors, Shanghai, China). The process of western blot was used as previously described [32]. The following primary antibodies were used: polyclonal anti-sEH antibody (Santa Cruz Biotechnology) and anti-GAPDH antibody (Sigma) was used as an internal loading control.

### Statistical analysis

All data are expressed as mean ± standard error (SEM). Differences of normal distributed continuous variables among groups were determined by unpaired *t*-test, while non-normal distributed continuous variables were compared by Mann-Whitney *U*-test. One way ANOVA and least-significant difference (LSD) test were also used for comparison among groups. A two-tailed P value < 0.05 was considered to be significant. Statistical analysis was done using SPSS version 17.0 (SPSS Inc., Chicago, IL, USA).

## References

[1] Tonelli M, Wiebe N, Culleton B, House A, Rabbat C, Fok M, McAlister F, Garg AX (2006). Chronic kidney disease and mortality risk: a systematic review. J Am Soc Nephrol.

[2] Yogi A, Callera GE, Tostes R, Touyz RM (2009). Bradykinin regulates calpain and proinflammatory signaling through TRPM7-sensitive pathways in vascular smooth muscle cells. Am J Physiol-Reg I.

[3] Zhang H, Wang T, Zhang K, Liu Y, Huang F, Zhu X, Wang MH, Tang W, Wang J, Huang H (2014). Deletion of soluble epoxide hydrolase attenuates cardiac hypertrophy via down-regulation of cardiac fibroblasts-derived fibroblast growth factor-2. Crit Care Med.

[4] Xu D, Li N, He Y, Timofeyev V, Lu L, Tsai HJ, Kim IH, Tuteja D, Mateo RK, Singapuri A, Davis BB, Low R, Hammock BD (2006). Prevention and reversal of cardiac hypertrophy by soluble epoxide hydrolase inhibitors. Proc Natl Acad Sci U S A.

[5] Zhang K, Wang J, Zhang H, Chen J, Zuo Z, Huang H (2013). Mechanisms of epoxyeicosatrienoic acids to improve cardiac remodeling in chronic renal failure disease. Eur J Pharmacol.

[6] Liu J, Zhou J, An W, Lin Y, Yang Y, Zang W (2010). Apocynin attenuates pressure overload-induced cardiac hypertrophy in rats by reducing levels of reactive oxygen species. Can J Physiol Pharmacol.

[7] Li YQ, Li XB, Guo SJ, Chu SL, Gao PJ, Zhu DL, Niu WQ, Jia N (2013). Apocynin attenuates oxidative stress and cardiac fibrosis in angiotensin II-induced cardiac diastolic dysfunction in mice. Acta Pharmacol Sin.

[8] Mouzaoui S, Djerdjouri B, Makhezer N, Kroviarski Y, El-Benna J, Dang PM (2014). Tumor Necrosis Factor-alpha-Induced Colitis Increases NADPH Oxidase 1 Expression, Oxidative Stress, and Neutrophil Recruitment in the Colon: Preventive Effect of Apocynin. Mediators Inflamm.

[9] Tain YL, Hsu CN, Huang LT, Lau YT (2012). Apocynin attenuates oxidative stress and hypertension in young spontaneously hypertensive rats independent of ADMA/NO pathway. Free Radic Res.

[10] Kim JH, Jang BG, Choi BY, Kim HS, Sohn M, Chung TN, Choi HC, Song HK, Suh SW (2013). Post-treatment of an NADPH oxidase inhibitor prevents seizure-induced neuronal death. Brain Res.

[11] Kinugasa S, Tojo A, Sakai T, Tsumura H, Takahashi M, Hirata Y, Fujita T (2011). Selective albuminuria via podocyte albumin transport in puromycin nephrotic rats is attenuated by an inhibitor of NADPH oxidase. Kidney Int.

[12] Ronco C, Haapio M, House AA, Anavekar N, Bellomo R (2008). Cardiorenal syndrome. J Am Coll Cardiol.

[13] Fujii H, Nishijima F, Goto S, Sugano M, Yamato H, Kitazawa R, Kitazawa S, Fukagawa M (2009). Oral charcoal adsorbent (AST-120) prevents progression of cardiac damage in chronic kidney disease through suppression of oxidative stress. Nephrol Dial Transplant.

[14] Panichi V, Maggiore U, Taccola D, Migliori M, Rizza GM, Consani C, Bertini A, Sposini S, Perez-Garcia R, Rindi P, Palla R, Tetta C (2004). Interleukin-6 is a stronger predictor of total and cardiovascular mortality than C-reactive protein in haemodialysis patients. Nephrol Dial Transplant.

[15] Zhang J, Chandrashekar K, Lu Y, Duan Y, Qu P, Wei J, Juncos LA, Liu R (2014). Enhanced expression and activity of Nox2 and Nox4 in the macula densa in ANG II-induced hypertensive mice. Am J Physiol Renal Physiol.

[16] Park YM, Park MY, Suh YL, Park JB (2004). NAD(P)H oxidase inhibitor prevents blood pressure elevation and cardiovascular hypertrophy in aldosterone-infused rats. Biochem Biophys Res Commun.

[17] Moe KT, Khairunnisa K, Yin NO, Chin-Dusting J, Wong P, Wong MC (2014). Tumor necrosis factor-alpha-induced nuclear factor-kappaB activation in human cardiomyocytes is mediated by NADPH oxidase. J Physiol Biochem.

[18] Imig JD (2012). Epoxides and soluble epoxide hydrolase in cardiovascular physiology. Physiol Rev.

[19] Elmarakby AA (2012). Reno-protective mechanisms of epoxyeicosatrienoic acids in cardiovascular disease. Am J Physiol Regul Integr Comp Physiol.

[20] Roman RJ (2002). P-450 metabolites of arachidonic acid in the control of cardiovascular function. Physiol Rev.

[21] Gauthier KM, Olson L, Harder A, Isbell M, Imig JD, Gutterman DD, Falck JR, Campbell WB (2011). Soluble epoxide hydrolase contamination of specific catalase preparations inhibits epoxyeicosatrienoic acid vasodilation of rat renal arterioles. Am J Physiol Renal Physiol.

[22] Pinto A, Abraham NG, Mullane KM (1987). Arachidonic acid-induced endothelial-dependent relaxations of canine coronary arteries: contribution of a cytochrome P-450-dependent pathway. J Pharmacol Exp Ther.

[23] Cerasola G, Nardi E, Palermo A, Mule G, Cottone S (2011). Epidemiology and pathophysiology of left ventricular abnormalities in chronic kidney disease: a review. J Nephrol.

[24] Zhang D, Xie X, Chen Y, Hammock BD, Kong W, Zhu Y (2012). Homocysteine upregulates soluble epoxide hydrolase in vascular endothelium *in vitro* and *in vivo*. Circ Res.

[25] Ai D, Pang W, Li N, Xu M, Jones PD, Yang J, Zhang Y, Chiamvimonvat N, Shyy JY, Hammock BD, Zhu Y (2009). Soluble epoxide hydrolase plays an essential role in angiotensin II-induced cardiac hypertrophy. Proc Natl Acad Sci U S A.

[26] Liu L, Chen C, Gong W, Li Y, Edin ML, Zeldin DC, Wang DW (2011). Epoxyeicosatrienoic acids attenuate reactive oxygen species level, mitochondrial dysfunction, caspase activation, and apoptosis in carcinoma cells treated with arsenic trioxide. J Pharmacol Exp Ther.

[27] Spector AA, Norris AW (2007). Action of epoxyeicosatrienoic acids on cellular function. Am J Physiol Cell Physiol.

[28] Liu Y, Liu X, Chen J, Zhang K, Huang F, Wang JF, Tang W, Huang H (2015). Apocynin Attenuates Cardiac Injury in Type 4 Cardiorenal Syndrome via Suppressing Cardiac Fibroblast Growth Factor-2 With Oxidative Stress Inhibition. J Am Heart Assoc.

[29] Goulitquer S, Dreano Y, Berthou F, Corcos L, Lucas D (2008). Determination of epoxyeicosatrienoic acids in human red blood cells and plasma by GC/MS in the NICI mode. J Chromatogr B Analyt Technol Biomed Life Sci.

[30] Zhang Y, Chan MM, Andrews MC, Mori TA, Croft KD, McKenzie KU, Schyvens CG, Whitworth JA (2005). Apocynin but not allopurinol prevents and reverses adrenocorticotropic hormone-induced hypertension in the rat. Am J Hypertens.

[31] Morgan LA, Olzinski AR, Upson JJ, Zhao S, Wang T, Eisennagel SH, Hoang B, Tunstead JR, Marino JP, Willette RN, Jucker BM, Behm DJ (2013). Soluble epoxide hydrolase inhibition does not prevent cardiac remodeling and dysfunction after aortic constriction in rats and mice. J Cardiovasc Pharmacol.

[32] Zhang K, Cheng G, Cai X, Chen J, Jiang Y, Wang T, Wang J, Huang H (2013). Malnutrition, a new inducer for arterial calcification in hemodialysis patients?. J Transl Med.

